# The Advancement-Rotation Principle in Chin Augmentation: A Preliminary Case Series

**DOI:** 10.1093/asjof/ojag110

**Published:** 2026-06-08

**Authors:** James Alexander Olding, Ashraf Messiha, Felix Wilhelm Karst, Nikola Kulik, Zeanab Chaer, Farhad B Naini

## Abstract

**Background:**

Injectable aesthetic treatments continue to grow in popularity globally. Hyaluronic acid–based filler remains the mainstay, used to augment or restore dimensions. Treatments to advance the chin must account for additional factors that are unique to this region, including the mandibular growth pattern, dental occlusion, and soft tissues in the form of the mentalis muscle.

**Objectives:**

This preliminary report synthesizes literature from across surgery, dentistry, and aesthetic medicine to propose an integrated method for chin assessment and treatment using injectables.

**Methods:**

We present a preliminary 3 patient case series drawing on surgical and nonsurgical cases. The presented integrated algorithm for chin assessment is applicable to medical aesthetics with the use of a smartphone camera.

**Results:**

Two nonsurgical patients each demonstrate retrognathia with contrasting mandibular growth patterns; one clockwise and one counter-clockwise. One surgical patient supports the presented methodology, where severe clockwise rotation of the mandible resulting from progressive condylar resorption was treated with 2-stage surgery.

**Conclusions:**

Effective lower face treatment requires integration of concepts from across surgery, dentistry, and aesthetic medicine. Facial growth patterns can be assessed clinically, enabling practitioners to plan and execute treatments in a way that promotes facial profile harmony and moderates an individual's aging trajectory. This preliminary series highlights how the growth rotation pattern and sagittal dimensions predictably impact lower facial features, offering novel treatment strategies. This article is the first to integrate concepts from Craniomaxillofacial surgery, Orthodontics, and Aesthetic Medicine into a chin treatment algorithm that can be easily implemented in medical aesthetics practice.

**Level of Evidence: 5 (Therapeutic):**

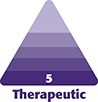

Research and historical study show that the importance of a harmonious and defined lower face transcends cultures and eras.^1^ Nonsurgical treatments may camouflage dimensional deficiencies in sagittal, transverse and vertical planes, as well addressing age-related changes through volume restoration. When approaching this area, the clinician must answer 2 key questions. The primary question concerns suitability for isolated chin treatment, which depends on assessment that includes an appraisal of the underlying skeletal and dental relationships. Attempts to advance the chin in isolation to mask a significant underlying sagittal mandibular deficiency, especially where this corresponds with a sagittal dental (incisal) discrepancy, will inevitably yield unsatisfactory results.^2^

If assessed as suitable for isolated chin treatment, the second question concerns the modality of choice. Options for augmenting the chin include surgical and nonsurgical, of which the most popular are surgical genioplasty, alloplastic implant placement, or injectable filler, which most commonly consists of hyaluronic acid (HA).^3,4^ All 3 modalities have been shown to be effective in the short to medium term, with better long-term results with surgical genioplasty, and a less invasive treatment with injectable filler, which requires reinjection sequentially to maintain results.^4,3^ Similar to surgical genioplasty, alloplastic implants are more invasive, requiring a surgical procedure. With growing evidence of implant-induced bone resorption at the mentum,^5^ coupled with a move away from placement of alloplastic material in the face, this is our least favored option. This modality is also commonly found to be used in cases of inappropriate masking of a dentofacial discrepancy, leading to unsatisfactory outcomes.

Providing facial profile harmony goes beyond purely augmenting dimensions. Mandibular rotation around the fulcrum of the temporomandibular joint (TMJ) and direction of osseous growth determine growth vectors during development.^6^ This also underpins our understanding of how certain craniomaxillofacial conditions develop in surgical practice.^6,7^ Effective diagnosis and treatment of complex craniofacial pathology requires interdisciplinary collaboration. This collaborative approach forms the basis of the present discussion, addressing a much-considered topic within aesthetic medicine, while drawing on principles from beyond medical aesthetics practice. To our knowledge, this article presents the first nonsurgical treatment approach to the chin that unifies evidence-based principles from across craniomaxillofacial surgery, craniofacial development, and aesthetic medicine. The approach presented is immediately applicable in everyday practice using a smartphone camera in clinic.

Craniofacial skeletal development is arguably the single most important factor in facial aesthetics and simultaneously one of the least understood.^8,9^ If we consider nonsyndromic patients with craniofacial anatomy that falls within the spectrum of populational diversity, without significant age-related changes, and with a body mass index (BMI) within normal range, the underlying etiology of facial profile disharmony will likely be linked to maxillary and mandibular development.

Facial profile harmony may be evaluated using a multitude of methods,^9,8^ with the facial angle of convexity being one such parameter commonly used.^4,10^ The facial angle is easy to measure and relatively reproducible; however, it does not indicate the etiology of profile disharmony and thus should be applied within a broader context. Developing a deeper understanding of chin aesthetics, it becomes clear that obtaining an accurate diagnosis for profile disharmony goes beyond linear measurements in sagittal or vertical planes. For example, the soft tissue pogonion may occupy the same sagittal position in 2 patients with very different diagnoses.^1,11^ Beyond linear measurements, chin form, centered around the labiomental fold and the aesthetic S-shape of the chin, is a key factor in profile analysis. This S-shape, or serpentine line, was described in 1753 by Hogarth as the line of beauty, signifying liveliness and activity, in contrast to straight or parallel lines.

In Craniomaxillofacial surgery, cephalometric analysis provides a more detailed overview of the etiology underpinning skeletal discrepancies that may manifest in dentofacial deformity.^9,12^ Using a standardized lateral cephalometric radiograph, we may evaluate sagittal and vertical relationships in the facial skeleton.^12^ These measurements are used in conjunction with facial assessment to treatment plan patients who present with dentofacial deformity, and who have opted to undergo surgery, commonly in addition to orthodontic treatment. It is clearly unfeasible and unethical to routinely expose patients seeking nonsurgical aesthetic treatments to ionizing radiation. There are nonetheless several clinical assessment methods that permit a more precise understanding of underlying craniofacial development without plain radiographs and which inform how this development could relate to the presentation of a patient in a medical aesthetics setting.^4,15^ (Figure 1)

**Figure 1.**
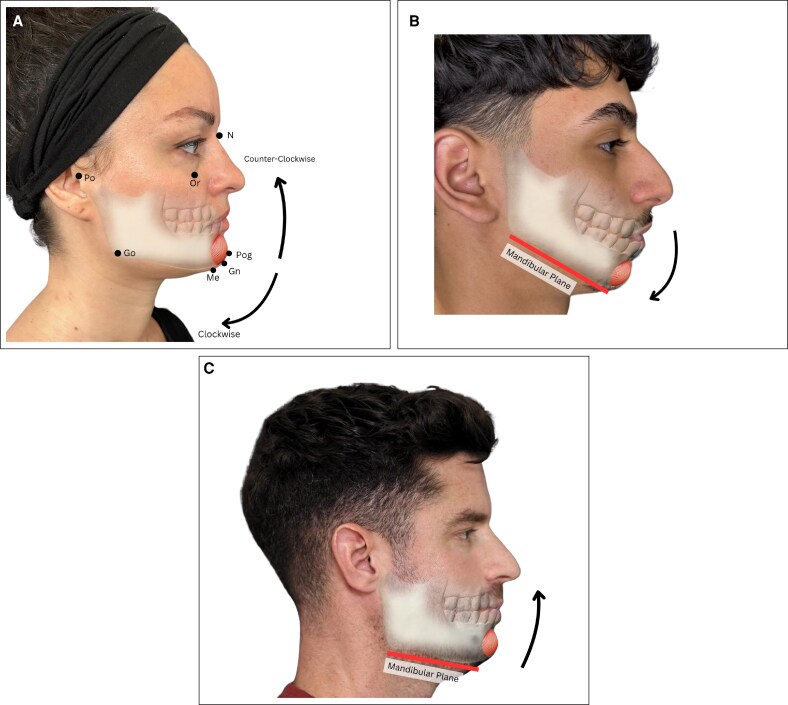
Standardized lateral views. (A) Female, 39 years old. Used for illustrative purposes. Soft tissue cephalometric points: Pog—Pogonion; Gn—Gnathion; Me—Menton; Go—Gonion; Po—Porion; Or—Orbitale; N—Nasion; (B) Male, 20 years old. Illustration of a high mandibular plane angle with clockwise rotation. (C) Male, 39 years old. Illustration of a low mandibular plane angle with counter-clockwise rotation.

The steepness or flatness of the plane running from the gonion to the menton in profile view, known as the Mandibular Plane (MP), provides information on the high, normal, or low angle nature of the patient's mandible.^6,14^ The reference for the mandibular plane is a horizontal plane, also known as the True Horizontal (TH), although some clinicians prefer the Frankfort Horizontal Plane. Using these planes, we can classify a patient as high (hyperdivergent, >32 degrees), normal, or low (hypodivergent, <22 degrees) angle.^12,13,15^

During growth, endochondral ossification at the condyle occurs in a superior direction, resulting in a downwards, forwards, and counter-clockwise movement of the jaw and chin. This occurs in coordination with vertical maxillary growth, leading to downward and forward facial growth relative to the cranial base. If there is a disruption to condylar growth (such as following illness or trauma), or where growth is unbalanced through greater downward vertical growth from the maxilla, condylar growth and chin point advancement become desynchronized.^13,6^

The result is a compensatory pattern of ossification, creating a posteriorly oriented vector, rather than a superiorly oriented one, leading to a clockwise (posterior) growth pattern, typified by a high angle, dolichocephalic phenotype, which tends to skeletal class 2.^6^ The actual sagittal growth of the mandible achieved will determine the skeletal and dental relationship upon growth completion. The growth direction resulting from the ossification pattern in the condyle will also impact features such as the antegonial notch, which is typically pronounced in the high angle, dolichocephalic, clockwise-growing phenotype.^13^

This is in contrast to counter-clockwise growth, where condylar growth is synchronous with, or exceeds, the downward growth of the maxilla. If unbalanced in favor of condylar growth, this results in a brachycephalic, or short face, and a chin point that rotates counter-clockwise, advancing with condylar growth.^14^ The final skeletal relation will depend on additional factors including the sagittal growth of the corpus, ramus, and mentum.^6,14,13^

Natural head position (NHP) can be defined as a standardized and reproducible orientation of the head in space when the subject is focusing on a distant point at eye level.^15^ As a physiological position distinct to each individual, it is widely accepted as the most accurate position in which to assess facial proportions, especially in patients with dentofacial discrepancies, who may commonly posture their head and/or jaw as camouflage.^1,15^ In contrast, the Frankfort Plane is an anatomical plane, chosen by anthropologists for use on human skulls. This plane has been shown to be divergent from the true horizontal plane across a spectrum of clinical presentations^1,15^ and subject to high individual variation. Despite ongoing common use of this plane among many facial plastic surgeons, our experience combined with the published literature supports the use of the NHP to derive true vertical and horizontal planes.

To obtain a lateral photograph in NHP, the patient should be standing or sat upright, looking straight ahead at a horizon, or alternatively at their own eyes in a mirror at their eye level.^15^ In our practice, we observe the patient from the moment they enter the room, and explain the need for a natural head position before photography. Where a patient is still posturing away from NHP, we ask them to look at an eye-level point in the distance.

Patients presenting with facial profile disharmony secondary to mandibular deficiency will usually present with either a Class 1 or Class 2 (Division 1 or 2) incisor relationship.^12^ Identifying the incisor classification will support or challenge the diagnosis of a patient's facial profile disharmony, for example in regard to the contribution of micrognathia or microgenia to the presentation, helping to guide and refine the treatment plan. It will also provide an opportunity to identify and refer onwards those patients who are unsuitable for primary nonsurgical treatments, such as those with a moderate to severe dentofacial finding.

Dental Class impacts facial aesthetics profoundly, both in its direct relationship to skeletal class, and in and of itself, especially where the incisors are considered.^15,9^ Any discussion on incisor classifications must first set out the distinction between sagittal (overjet) and vertical (overbite) relationships between the upper and lower central incisors. While a comprehensive discussion of dental classifications is outside the scope of this article, it is crucial that all injectors are able to recognize dental class in patients presenting with facial profile disharmony.

## METHODS

We present an initial 3-patient series (2 nonsurgical, 1 surgical) to demonstrate the advancement-rotation principle, and its application to chin treatment in aesthetic medicine. The current discussion is focused on disharmony due to a retropositioned (regtrogenic) or small (microgenic) chin. It is important to note that retrogenia and microgenia are not the same, although may coexist. Retrognathia (retropositioned jaw) and micrognathia (small jaw) may also exist with a small or retropositioned chin, though again each can occur independently.

The interdisciplinary principles and parameters discussed in this article are well-established. However, the integrated approach set out, including using a smartphone or device, is novel in medical aesthetics. Consideration for nonsurgical treatment to the lower face should be reserved for patients with mild sagittal dental and/or skeletal discrepancies. Moderate-to-severe dentofacial deformity can be defined according to the Index of Orthognathic Functional Treatment Need (IOFTN), representing skeletal and occlusal discrepancies of sufficient severity to warrant consideration of orthognathic treatment (Grades 4 and 5).^16^ After excluding patients with moderate-to-severe dentofacial deformity, suspected condylar pathology, any functional issue, or unrealistic expectations around treatment, patients can be photographed in profile to aid diagnosis and planning.

Key parameters:

- True Vertical (TV): a vertical line drawn parallel to a plumb line hanging from the ceiling.
- True Horizontal (TH): derived from a line drawn horizontally across the profile perpendicular to the TV, and parallel to the floor with the patient in NHP^15^
- The Mandibular plane (MP): This is a line connecting the menton and the gonion, running along the lower border of the mandible.^12^
- True Horizontal to Mandibular Plane Angle (TH-MP): The intersection of the TH and the MP, which informs the nature of the bite angle; this being high, normal or low angle. It should be noted that the Frankfort Plane and TH plane may be identical or near identical.^15^ On clinical assessment and photography review, the point at which the TH and MP intersect in relation to the occiput can provide a gross assessment of the angle.

The TH-MP can be traced clinically, without the need for radiographs. Using a patient photograph in profile allows a quick and reproducible evaluation of 2 key features:

- The sagittal (AP) relationship of the mandible and chin (informing skeletal relationship^1,2^)
- The vector of growth of the mandible (high angle—clockwise, low angle—counter-clockwise^6,12^)

### Ethics

This study represents a retrospective review of patients treated as part of routine clinical care. No alterations to treatment protocols were made for research purposes, and no additional interventions or investigations were undertaken. All data analyzed were derived from standard-of-care management. The study was conducted in accordance with the principles of the Declaration of Helsinki. Written informed consent for publication of clinical data and images was obtained from all patients included in this report.

## RESULTS

### Case 1—Nonsurgical: High Angle

**Figure 2.**
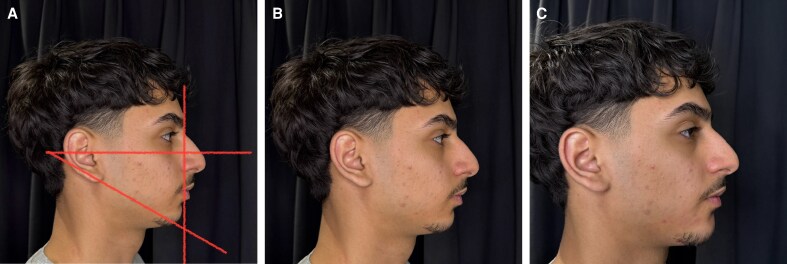
Male, 20 years old. (A) Intersection of the TH and MP lines anterior to the occiput indicates a high angle presentation. (B) Pretreatment. (C) Immediately posttreatment. Treatment provided: 1 mL Juvederm Volux with Lidocaine (VYC-25L Hyaluronic Acid Filler) (supraperiosteal at Gnathion), 2 mL Juvederm Voluma with Lidocaine (VYC-20L Hyaluronic Acid Filler) (superficial at Labiomental crease and Gnathion), 1 mL VYC-25L (anterior mandibular border to Menton in subcutaneous plane). Total of 4 mL over 1 session.

Clockwise Mandibular Growth pattern in a 20-year-old male presenting with borderline high angle mandibular plane angle (Figure 2). He demonstrates mandibular retrognathia corresponding with a Class II division 2 incisor relationship and mild skeletal 2 base. He presents with a pleasant labiomental crease and appropriate submental-cervical angle. This case was approached with balanced deep and superficial HA placement to provide chin point advancement while controlling the rotation of the mentalis and pogonion; deep placement on to bone to promote counter-clockwise rotation of mentalis, and superficial placement to preserve the pleasant labiomental crease and promote clockwise rotation of mentalis. Sagittal splinting of the subcutaneous soft tissues was also undertaken along the lower border of the anterior mandible toward the menton.

### Case 2—Nonsurgical: Low Angle

**Figure 3.**
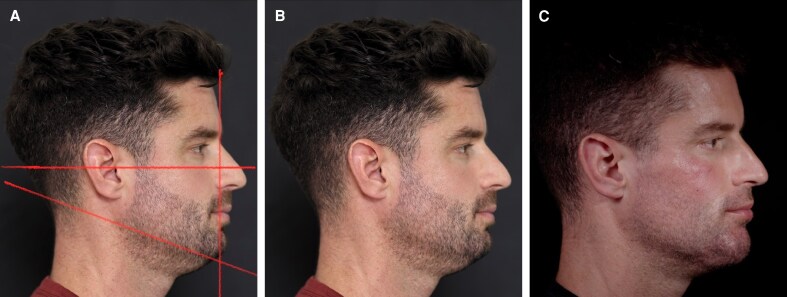
Male, 39 years old. (A) Intersection of the TH and MP lines posterior to the occiput indicates a low angle presentation. (B) Pretreatment. (C) Two months after first treatment, immediately after second treatment. Treatment provided: 2 sessions, 2 months apart, with each treatment consisting in 2 mL VYC-25L (superficial at labiomental crease and gnathion), 2 mL VYC-25L (anterior mandibular border to Menton in subcutaneous plane). Total of 8 mL over 2 sessions.

Counter-clockwise mandibular growth pattern in a 39-year-old male presenting with a mildly low angle mandibular plane (Figure 3). He presents mandibular retrognathia with mild microgenia, corresponding with a Class II division 2 incisor relationship, with mild-to-moderate skeletal 2 base. Features include a high-riding, hyperkinetic and counter-clockwise rotated mentalis, obtuse cervicomental angle, and deep labiomental crease. This case was treated with an exclusively superficial approach, with HA placement aimed at myomodulating mentalis, and clockwise rotating the pogonion, which simultaneously increases the apparent lower face height, which is advantageous in low angle cases. Sagittal splinting of the subcutaneous soft tissues was also undertaken along the lower border of the anterior mandible toward the menton. Given the degree of camouflage required, treatment was repeated after 2 months, providing adequate time for soft tissue accommodation and optimizing patient comfort.

### Case 3—Surgical: Progressive Condylar Resorption

Progressive condylar resorption (PCR) is a condition that affects females 10 times more frequently than males, with a predilection for adolescents and young adults.^17^ The temporomandibular joint (TMJ) contains estrogen receptors, and there is ongoing debate around the role of hormones in the pathogenesis of this condition, and other conditions such as temporomandibular joint dysfunction.^17^ Progressive condylar resorption may be referred to as “cheerleader syndrome,” given the demographic associations and the proposed link with repeated microtrauma to the condyle over time, of as part of a distinct injury. Other important associations include inflammatory conditions such as Juvenile idiopathic arthritis, rheumatoid arthritis, psoriatic arthritis, and previous surgery or orthodontics. Some patients have no identifiable factor, underpinning the interchangeably used term idiopathic condylar resorption (ICR).^18^

**Figure 4.**
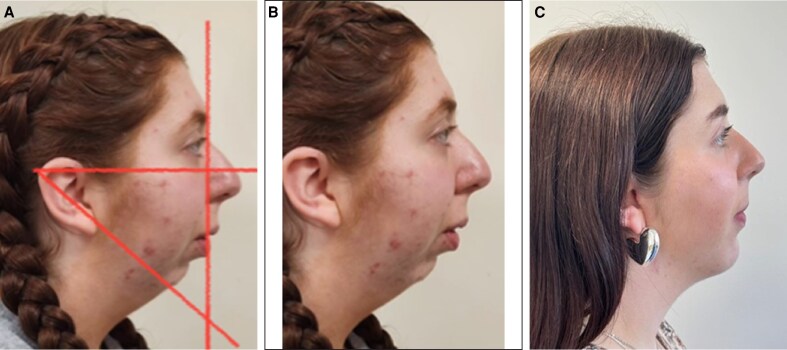
Female, 18 years old. (A) Progressive condylar resorption case, with demonstration of preoperative True Horizontal (TH) and True Vertical (TV) marked with patient in natural head position (NHP). A high angle mandibular plane is demonstrated with clockwise rotation of the pogonion position. Note the divergence of the TH from the Frankfort Plane. (B) Pretreatment. (C) Postoperative views following a 2-staged approach of surgical genioplasty followed 6 months later by concomitant maxillary advancement with bilateral TMJ replacements. Image taken 12 months after image B.

Our experience of surgically treating over 100 cases of condylar resorption over the past 10 years has led us to take a staged surgical approach, consisting in a genioplasty first, followed by concomitant TMJ replacements and Le Fort I osteotomy second, typically 6 to 12 months later. The soft tissue expansion that a staged approach offers, especially in regard to the mentalis and perioral musculature, is a key advantage. Our experience provides a unique insight into the behavior of the lower facial soft tissues resulting from distinct and significant dimensional alterations which is transferrable to nonsurgical practice. (Figure 4)

The treatment objectives include the restoration of vertical and sagittal dimensions, and crucially, the counter-clockwise rotation of the chin point. This advancement-rotation addresses both the functional and aesthetic issues that these cases exemplify, with highly applicable principles to the much smaller dimensional adjustments we make in medical aesthetics through camouflage and myomodulation.

## DISCUSSION

### Myomodulation and Mentalis Activity

Understanding how to approach mentalis is the key to achieving optimal chin treatment outcomes nonsurgically.^9,20^ Importantly, the motivations for seeking to modulate mentalis activity and adjust the rotational position of the pogonion should not overlook the underlying etiology, which may be skeletal and developmental.^8,19^ As such, we have the symptom (skin puckering, or an aberrant pogonion position) and the cause (mandibular growth pattern and linear dimensions). It should be added that other factors such as skin and dental factors also play a key role, which increase in importance with aging.^19,20^

Myomodulation can be described as the modulating effect of filler on muscle activity, and in medical aesthetics, it may be utilized to address muscle imbalances linked to age-related volume loss (eg, deep medial cheek fat and zygomaticus major activity), or to diminish developmental muscle overactivity (eg, gingival smile).^19^ This concept is commonly discussed in relation to the mentalis and can be effectively employed to down modulate the activity of mentalis. Nonetheless, there are some key differences around the treatment of mentalis vis-à-vis other mimetic muscles.

First, mentalis runs from deep (incisive fossa of mandible) to superficial (dermal insertion at the pogonion) in a short, oblique manner; this means that the principles relevant to deep or superficial injection of other longitudinal mimetic muscles do not necessarily apply in the same way to mentalis, especially where the advancement-rotation principle is not adequately considered.^2,22,21^ Second, the insertion of mentalis is at the soft tissue apex of the lower jaw, which itself forms a triangle that hinges and translates open and closed. Consequently, mentalis is an anatomical extension of the mandible toward the skin of the gnathion and pogonion, and will behave in a predictable way based on both growth rotation pattern and sagittal skeletal dimensions.^22,21,9^

We suggest that discussion of mentalis in medical aesthetics has hitherto been limited by not considering these concepts of facial growth and mandibular rotation. A failure to stratify patients with chin retrusion based on their growth rotation pattern has likely limited the findings in a number of previous studies, including those assessing the impact of different injection depths on appearance and mentalis activity.^22,21^

### Advancement Through Rotation

Surgically and nonsurgically, sagittal chin changes are achieved through rotation and correspondingly rotation will produce sagittal changes.^6,11,7^ These 2 cause and effect relationships must be considered with every chin treatment; they are inextricably linked. Thinking solely or primarily about advancement, without accounting for rotation, will yield suboptimal outcomes and may result in undesired effects.^6^

**Figure 5.**
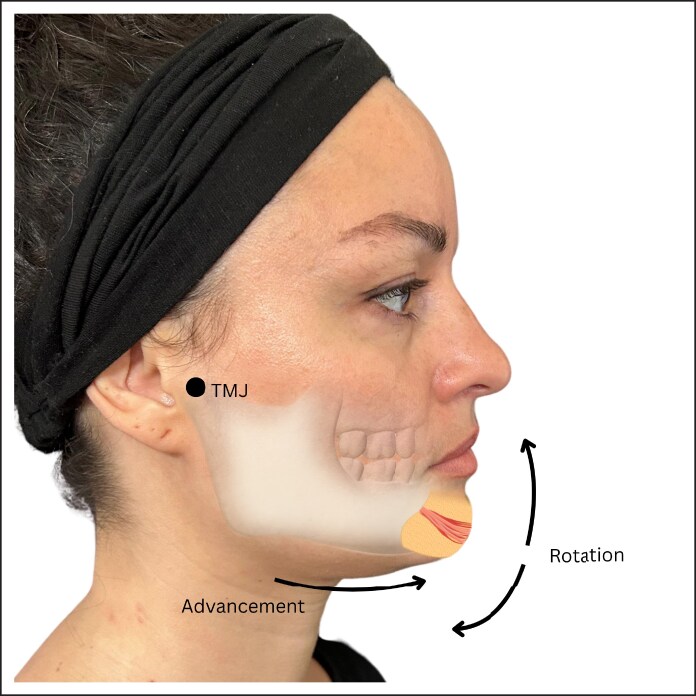
Female, 39 years old, image used for illustrative purposes. The 2 primary considerations when seeking chin augmentation; sagittal advancement, and mentalis rotation in a clockwise or counter-clockwise direction.

We can utilize a patient's growth rotation pattern to advance or treat the chin more effectively and aesthetically, predicting how superficial or deep placement will impact rotation of the mentalis and pogonion, as summarised in Table 1. The TH-MP informs the growth rotation pattern of the mandible, which in turn will inform the most appropriate treatment strategy for advancing the chin.^15,23^ The overarching consideration is of the desired rotational, or clockwise, position of mentalis and the pogonion. We propose that the treatment objective should purposefully consist in sagittal advancement combined with one of the following 3 options:

1. Preservation of rotational position, with maintenance of the current labiomental fold.
2. Clockwise rotation, with softening of the labiomental fold and increase in apparent vertical dimension.
3. Counter-clockwise rotation, with pronunciation of the pogonion and creation/deepening of the labiomental fold (Figure 5).

**Table 1.** Nonsurgical Treatment Strategies Based on Assessment. TH-MP, True Horizontal to Mandibular Plane Angle.

	Rotation aim	Injection	Additional
Normal TH-MP	Neutral	Balanced Deep + Superficial	Care to preserve and enhance chin features like labiomental crease and aesthetic “S” shape
Low TH-MP	Clockwise	Superficial predominates	Consider perioral treatment early. Consider treatment to open the nasolabial angle.
High TH-MP	Counter-clockwise	Deep predominates	Perform chin soft tissue pinch test to assess for ability to accommodate injected volume. Assess oral competency and lower lip protrusion. Assess chin-neck distance and exclude progressive or underlying cause like progressive condylar resorption (PCR) or obstructive sleep apnea (OSA)

### Predicting Lower Face Aging

Understanding a patient's growth pattern can provide an opportunity to intervene proactively. Classical aging involves progressive counter-clockwise rotation of the mandible about the TMJ.^24,19^ This is driven by progressive bone resorption, loss of clinical crown height of teeth through attrition, and soft tissue changes affecting the chin and perioral region.^24,8^ As such, low TH-MP, forward growth patterns present greater initial challenges.^14^ If aging is represented by counter-clockwise movement from 6 O'clock backwards, then it is obvious that having a genetic starting point of 5 O'clock requires more prompt intervention. Treatments should be aimed at preserving lower face height, opening the nasolabial angle and preserving a shallow labiomental crease. Perioral treatment to address muscle imbalance and provide structure and projection is also key. Overall, a thorough understanding of the interplay between skeletal, dental, and soft tissue changes will enable optimal diagnosis and treatment over years.^8,19^

Patients with a high mandibular plane angle and increased lower anterior face height present different challenges.^25^ Treated surgically, these patients benefit from approaches that project the chin sagittally, achieved through a combination of counter-clockwise rotation of the mandible, with sagittal advancement.^25^ Nonsurgically, the same principles should be applied, using HA-filler deep to mentalis to rotate the pogonion counter-clockwise, while splinting the submental tissues to project the soft tissue gnathion anteriorly. Identifying the chin landmarks accurately can be more challenging in this group, especially where retrognathia is associated with microgenia.^14^

Finally, part of holistic wellness and positive aging must involve a broader clinical approach, identifying issues that may exacerbate aging. These include common conditions like bruxism, which can accelerate dental attrition leading to unfavorable occlusal changes, and obstructive sleep apnea (OSA), which is associated with micrognathia and can lead to a host of deleterious physiological effects.^12,16^

## CONCLUSIONS

1. Chin rotation and sagittal advancement are inherently linked—the advancement-rotation principle is presented in this article for the first time in medical aesthetics literature
2. Using a smartphone or tablet, clinicians can easily trace a patient's true horizontal and mandibular planes, facilitating a more precise appraisal of the growth pattern
3. The mentalis muscle is a unique mimetic muscle, and its behavior is intrinsically linked to growth pattern of the mandible
4. Previous studies of mentalis have been limited by the failure to stratify patients based on growth pattern
5. Integrating the presented concepts into medical aesthetics practice allows more effective chin treatment, reduced unwanted effects, and a method for predicting an individual patient's aging trajectory which can be modified through prejuvenation.

### Limitations

This is a preliminary case series with few cases, serving to outline the synthesis of concepts presented. Future studies should incorporate a larger series of patients with statistical analysis of outcomes. The suggested injection volumes and treatment approaches are provided as references; further research is warranted to provide validation of the treatment approach. While this discussion is applicable to older patients, the bony changes that occur throughout life must be considered as potentially masking the growth pattern that an individual will best demonstrate on completion of adult growth, usually toward the end of the second decade. This discussion focuses on assessment and treatment of sagittal discrepancies primarily, rendering it less relevant for guiding treatment in other dimensions. For example, a common concern for patients as they age is the pre-jowl sulcus; incorporating this area into our approach would require a more comprehensive integration beyond sagittal analysis.
